# Exercise as a Promising Adjunct Treatment for Methamphetamine Addiction: Advances in Understanding Neuroplasticity and Clinical Applications

**DOI:** 10.3390/brainsci15121339

**Published:** 2025-12-16

**Authors:** Yongting Li, Xiaolong Chen, Tingting Wang, Wanlin Zou, Yong Tang, Zhigang Li

**Affiliations:** School of Physical Education, Southwest Medical University, Luzhou 646000, China; 18226377721@163.com (Y.L.); 13731231813@163.com (X.C.); w18148063587@163.com (T.W.); zouwanlin1994@126.com (W.Z.); t15908365447@163.com (Y.T.)

**Keywords:** methamphetamine use disorder, exercise interventions, neuroplasticity, relapse prevention, clinical translation

## Abstract

**Background:** Methamphetamine (Meth) addiction, with its high relapse rates, poses a significant global challenge. Conventional therapies remain inadequate, highlighting the need for effective adjunctive treatments. **Objective:** This review synthesises evidence to propose a novel ‘Exercise Modality–Neural Target–Rehabilitation Stage’ integration model, elucidating how aerobic, resistance, and mind–body exercises differentially target specific neural pathways to ameliorate cognitive deficits, emotional dysregulation, and craving in Meth use disorder. **Methods:** A narrative synthesis of 84 studies (up to March 2025) from PubMed, Web of Science, and CNKI was conducted, focusing on the neurobiological basis and clinical application of exercise interventions. **Results:** The analysis identifies a key overarching neurobiological pattern: different exercise modalities work complementarily to reverse Meth-induced imbalance in glutamate/gamma-aminobutyric acid (Glu/GABA) neurotransmitter homeostasis. Aerobic exercise upregulates prefrontal–striatal BDNF to enhance cognitive control, while resistance training modulates the amygdala–striatal dopamine system to improve emotional stability. Additionally, mind–body exercises help balance the autonomic nervous system, which in turn helps manage cravings. Building on this, we construct a standardised ‘screening–assessment–prescription’ framework to guide personalised interventions across the various stages of withdrawal. **Conclusions:** The primary contribution of this review is the integrative model that positions exercise as a precise, evidence-based rehabilitation strategy. The proposed framework provides a practical blueprint for clinical translation, with future research focusing on developing personalised intelligent rehabilitation systems by integrating multimodal exercise with advanced technologies.

## 1. Introduction

Meth abuse constitutes a severe global public health challenge, with the illicit drug user population reaching 292 million in 2022—a 20% increase over the past decade [1]. The chronic and relapsing nature of Meth use disorder stems from complex neuroadaptations that disrupt brain homeostasis, presenting a formidable obstacle to sustained recovery [2]. This challenge is particularly pressing in specific regions; for instance, in China, Meth is classified as a Class I psychotropic substance and accounts for 51% of the current drug-using population, underscoring the urgent need for effective interventions given its significant social harm [3,4].

At the neurobiological level, Meth use disorder is characterised by a profound dysregulation of key neurotransmitter systems, primarily involving the dopamine (DA) and glutamate Glu pathways [5]. This dysregulation leads to core clinical manifestations, including cognitive impairment, emotional dysregulation, and social dysfunction [6]. Chronic Meth exposure not only impairs dopaminergic transmission but also induces widespread neural circuit dysfunction, which elevates the risk of cardiovascular and cerebrovascular pathologies [7]. These symptoms are frequently compounded by psychiatric comorbidities, resulting in significant impairments in social and occupational functioning, alongside substantial socioeconomic burdens on individuals, families, and broader societal systems [8,9]. Current treatments remain inadequate: opioid-based therapies risk iatrogenic dependence [10], while psychological interventions are protracted and variably effective [11]. This critical gap between mechanistic understanding and clinical application highlights the necessity for safe, effective, and scalable non-pharmacological adjunctive treatments that precisely target the core pathophysiology of Meth use disorder. In this context, physical exercise has emerged as a promising therapeutic modality. Its pleiotropic effects engage multiple physiological systems, offering a holistic approach to rehabilitation [12].

Globally, exercise intervention is increasingly recognised as a core component in substance use disorder rehabilitation [13]. As an innovative approach, it simultaneously improves physical and mental health while reducing relapse rates, establishing a cost-effective adjunct therapy [14,15,16]. Previous reviews and meta-analyses confirm the general benefits of exercise for Meth use disorder, particularly for craving, cognitive function, and emotional state [17,18,19]. However, the existing evidence remains fragmented. While clinical studies affirm the efficacy of structured exercise, translating these findings into precise clinical practice is challenging. Prior reviews often offer a generalised perspective, lacking a systematic synthesis of how distinct exercise modalities (e.g., aerobic, resistance, mind–body) differentially target the specific neurobiological deficits underlying cognitive, emotional, and craving symptoms in Meth use disorder. Moreover, an overarching conceptual framework linking these mechanistic insights to personalised, stage-specific rehabilitation protocols is absent.

To address this gap, this narrative review proposes an integrative ‘Exercise Modality–Neural Target–Rehabilitation Stage’ model. By synthesising evidence on Meth addiction mechanisms and exercise-induced neuroplasticity, it aims to: (1) elucidate the distinct neurobiological mechanisms through which different exercise modalities counteract specific pathological processes; and (2) establish a refined, evidence-based framework to optimise intervention timing and prescription design across the recovery continuum. The primary contribution is positioning exercise not merely as a general wellness aid, but as a precise, mechanism-informed rehabilitation strategy. As a narrative synthesis, this review prioritises mechanistic depth and theoretical framework construction over exhaustive evidence enumeration, aiming to generate testable hypotheses for future research and clinical applications.

## 2. Methods

### 2.1. Review Scope and Theoretical Foundation

Aiming to develop a coherent framework for exercise intervention in Meth use disorder, this review is guided by three specific research questions: (1) What are the distinct neurobiological mechanisms through which different exercise modalities (aerobic, resistance, mind–body) alleviate specific symptoms of Meth use disorder? (2) How can these mechanistic insights be translated into personalised exercise prescriptions across different stages of rehabilitation? (3) What is the role of socio-environmental factors in moderating the effectiveness of exercise interventions? To address these questions, the review is structured around the proposed ‘Exercise Modality–Neural Target–Rehabilitation Stage’ model, which serves as the primary theoretical lens for synthesising evidence. This section articulates these goals and clarifies the scope of the inquiry, laying the necessary groundwork for the mechanistic and clinical synthesis presented in the subsequent sections.

### 2.2. Literature Search and Study Selection Process

**Figure 1:**
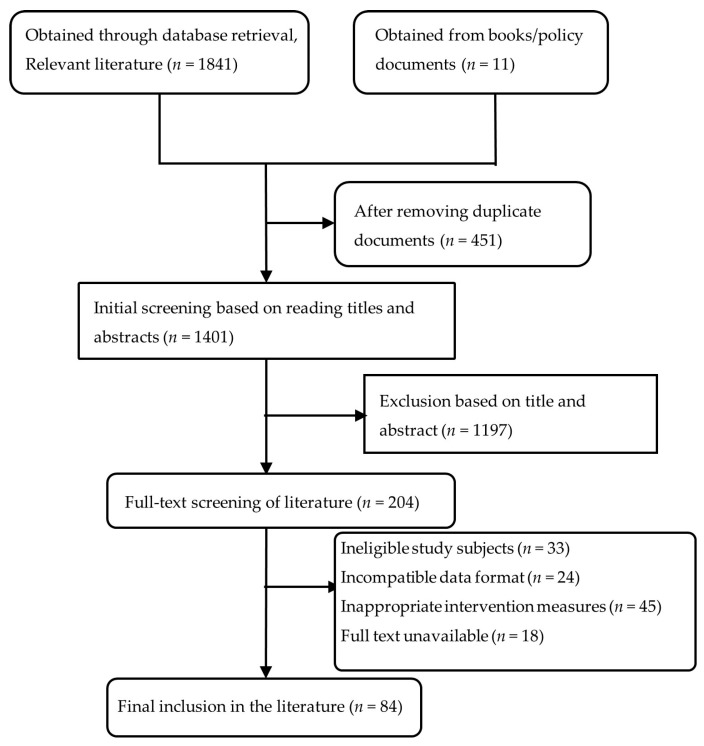
Literature Screening Flowchart.

To establish a comprehensive evidence base, the literature search prioritised studies on exercise interventions for Meth use disorder while also incorporating seminal reviews and original articles on its neurobiological and social mechanisms. Searches were conducted in multiple databases (PubMed, Web of Science, CNKI, and grey literature) up to March 2025. The search strategy employed a combination of keywords and Medical Subject Headings (MeSH) terms related to three conceptual groups: (1) the population (e.g., ‘methamphetamine’ OR ‘Meth’ OR ‘amphetamine-related disorders’); (2) the intervention (e.g., ‘exercise’ OR ‘physical activity’ OR ‘training’ OR ‘aerobic’ OR ‘resistance training’ OR ‘yoga’ OR ‘tai chi’); and (3) the outcomes/context (e.g., ‘addiction’ OR ‘craving’ OR ‘relapse’ OR ‘neuroplasticity’ OR ‘rehabilitation’). These groups were combined using the Boolean operator ‘AND’. The study screening process is detailed in Figure 1. Inclusion criteria covered: (1) original research and review articles; (2) human or relevant animal models of Meth use disorder; (3) interventions involving structured physical exercise; and (4) outcomes related to craving, cognitive function, emotional state, relapse rates, or neurobiological mechanisms. Studies were excluded if they were not Meth-related, lacked an exercise intervention protocol, or had full-text accessibility issues. The screening process was conducted independently by two authors based on titles and abstracts, followed by a full-text review of potentially eligible studies. Any disagreements were resolved through discussion or, if necessary, by consultation with a third author.

### 2.3. Data Extraction and Synthesis Method

The narrative synthesis followed an iterative, two-phase process. A standardised data extraction form was used to catalogue key information from each included study, including authors, publication year, study design, participant characteristics, detailed exercise parameters (modality, frequency, intensity, and duration), primary outcomes, and key mechanistic or efficacy findings.

#### 2.3.1. Phase 1: Categorisation and Within-Modal Analysis

Studies were first categorised by primary exercise modality (aerobic, resistance, or mind–body). Within each category, we identified the proposed neurobiological mechanisms and primary outcomes.

#### 2.3.2. Phase 2: Cross-Modal Comparison and Model Development

A comparative analysis was then conducted across the categories to identify convergent patterns (e.g., complementary restoration of Glu/GABA homeostasis), divergent findings, and critical evidence gaps. This analysis specifically sought patterns of convergence, divergence, and gaps, which enabled the inductive development of the overarching ‘Exercise Modality–Neural Target–Rehabilitation Stage’ model, ensuring it was grounded in the evidence.

Based on this synthesis, 15 highly relevant core papers were selected from the 84 included studies for detailed presentation and discussion. The selection criteria prioritised methodological quality (e.g., RCTs), clarity in illustrating a specific mechanism or application, and relevance to the integrative model. To balance conciseness with depth, core information from 10 papers is summarised in Table 1, while complete details for all 15 are provided in Supplementary Table S1.

## 3. Meth Mechanisms of Meth Use Disorder

Meth use disorder involves complex multi-system mechanisms, rooted in neurobiological adaptations, while significantly modulated by socio-environmental factors. This section first delineates the neurobiological mechanisms, analysing Meth’s detrimental effects on the central nervous system across molecular, cellular, and neural circuit levels. It then examines key socio-environmental factors—such as social support, living conditions, and peer influence—in the onset and maintenance of the disorder. Together, these analyses establish an integrated mechanistic foundation for developing the exercise intervention strategies discussed subsequently.

### 3.1. Neurobiological Mechanisms

The addictive properties of Meth are fundamentally driven by cross-circuit pathological remodelling resulting from an imbalance between the DA and Glu systems. As a key neurotransmitter in the reward pathway, DA is synthesised in the ventral tegmental area (VTA) and projected via neural fibres to limbic nuclei, including the nucleus accumbens (NAc), amygdala (AMY), prefrontal cortex (PFC), and hippocampus (HIPP). The spatiotemporally specific release of DA forms the neural coding basis for reward effects [28,29]. Meth competitively inhibits the dopamine transporter (DAT), triggering two effects: it blocks the reuptake of synaptic DA. It reverses the transport direction of DAT, thereby inducing the abnormal release of presynaptic DA into the NAc [6]. The persistent accumulation of DA leads to hyperactivation of the mesolimbic reward pathway, inducing synaptic receptor density and other neuroplastic changes in Nac neurons. These alterations ultimately result in abnormal sensitisation to Meth stimulation, forming the molecular basis for the consolidation of addictive behaviours.

At the circuit level, Glu signalling mediates Meth addiction progression through three key mechanisms: First, AMPA/NMDA receptor activation in the NAc triggers neuronal depolarisation and dendritic spine remodelling, integrating inputs from the AMY, PFC, and HIPP to transform environmental cues into drug-seeking motivation [30]. Second, VTA DA neurons receive Glu projections from the hypothalamus and medial PFC; metabotropic Glu receptors on these neurons fine-tune DA release thresholds in the NAc and PFC [31]. Third, presynaptic vesicular Glu transporters regulate Glu concentration, acting as a molecular switch for relapse behaviour after abstinence [32]. Meth further disrupts this balance by suppressing GABAergic inhibitory control while enhancing excitatory Glu input to the VTA, thereby amplifying DA release and perpetuating a cycle of reward reinforcement, cue-induced craving, and compulsive use [33].

**Figure 2:**
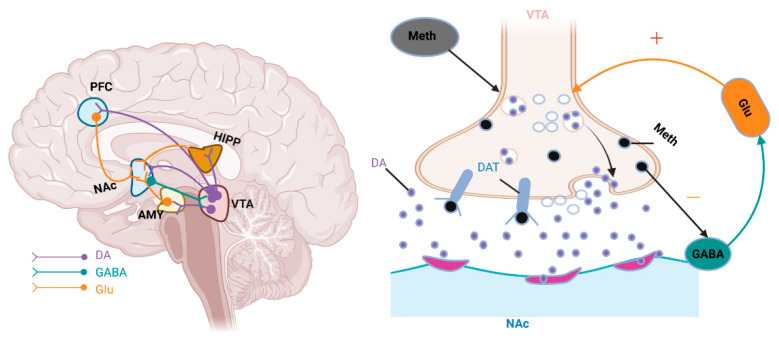
Schematic diagram of neurobiological mechanisms underlying Meth use disorder.

Chronic Meth exposure also induces cross-system damage through oxidative stress, excitotoxicity, and epigenetic reprogramming, resulting in widespread impairment of DA, Glu, and serotonin receptors [20]. These neurobiological abnormalities, modulated by socio-environmental factors, establish a dual foundation for biopsychosocial intervention strategies. Critically, the severity of impairment is dose- and duration-dependent: chronic high-dose use causes profound DA terminal degeneration, Glu/GABA imbalance, and cognitive deficits, whereas short-term low-dose exposure leads to reversible synaptic changes. This dose–response gradient directly influences clinical symptoms and the potential for exercise-induced neuroplasticity recovery, underscoring its importance in designing personalised rehabilitation protocols. Figure 2 summarises these neurobiological pathways in Meth use disorder.

### 3.2. Social Environment

Beyond the neurobiological mechanisms, the effectiveness of exercise interventions is also significantly moderated by the social environment. The social climate critically influences the development of Meth use disorder, with socioeconomic status, family support, and peer influence being primary contributors. Individual risk factors, such as impulsivity and novelty-seeking tendencies, are recognised as early behavioural markers [34,35]. Knerich et al. [36] found that this complexity is highlighted by findings from marginalised communities, where residential proximity and literacy levels shape distinct drug-use networks. Interestingly, the role of family income appears nuanced. At the same time, a low-income environment is a known risk factor for adolescents [37]; it is also associated with other factors that may mitigate its impact. Conversely, a positive family environment, adequate social support, and a healthy lifestyle can act as protective factors, reducing the likelihood of addictive behaviours in adolescents [38]. However, Marzban et al. [39] found that adolescents from high-income families were more likely to use drugs compared to those from low-income households, speculating that this may be attributed to greater resource availability for acquisition and experimentation among affluent youth. These findings collectively demonstrate that Meth use disorder is dynamically influenced by multi-level factors spanning individual traits, family, community structure, and subcultural elements. Consequently, single-dimensional interventions are inadequate, underscoring the necessity for a comprehensive ‘biological–psychological–social’ prevention and control system [40,41].

## 4. Exercise Interventions for Meth Use Disorder

Given that the occurrence and progression of Meth use disorder are closely associated with neurobiological changes and social environmental factors, exercise, as a safe and easily implementable non-pharmacological intervention, has demonstrated potential value in the rehabilitation of Meth use disorder [42]. This section reviews the application of exercise interventions for Meth use disorder, focusing on analysing the internal mechanisms through which exercise exerts its effects, exploring the impacts of different exercise types on craving in Meth-dependent individuals, and elaborating on the role of exercise in improving psychosocial functioning among Meth abstainers and enhancing the physical health of patients with Meth use disorder.

### 4.1. Mechanisms of Action of Exercise Intervention in Meth Use Disorder

**Figure 3:**
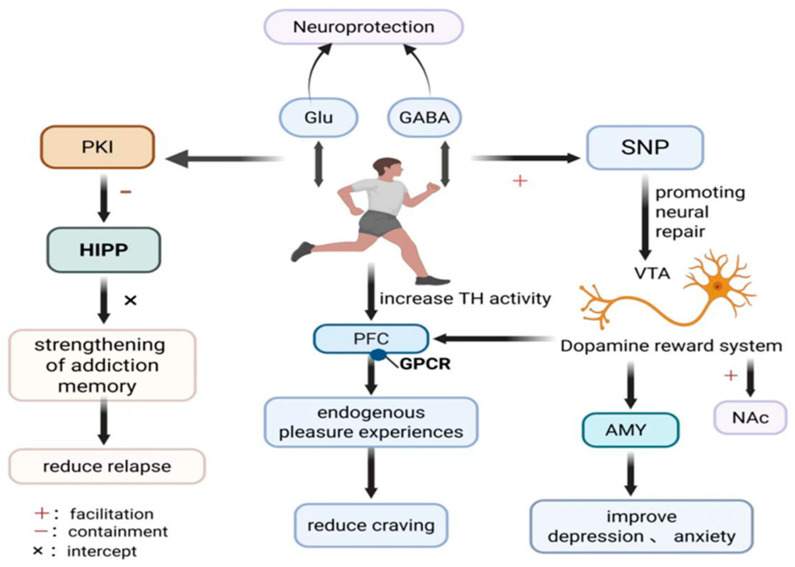
Exercise Intervention Mechanisms Chart.

This section comprehensively explains the synergistic effects through which exercise exerts therapeutic benefits via multidimensional neurobiological mechanisms. While previous reviews (e.g., Morais et al. [43]) have outlined the potential neurobiological benefits of exercise for Meth use disorder, the specific mechanistic pathways and their integration into a comprehensive therapeutic model remain underexplored. This review addresses this gap by systematically elaborating on the multi-dimensional neurobiological mechanisms through which exercise exerts therapeutic effects on Meth withdrawal. Specifically, exercise upregulates the activity of tyrosine hydroxylase (TH) in the VTA, thereby promoting DA synthesis and enhancing the expression of DA receptor G protein-coupled receptors (GPCRs). This mechanism partially activates the mesolimbic DA reward pathway, replacing exogenous Meth-induced reward with endogenous pleasurable experiences, consequently attenuating craving during withdrawal periods [14,44]. This reward-substitution effect is further supported by evidence from both acute and chronic aerobic exercise interventions. For instance, Saanijoki et al. demonstrated that moderate aerobic exercise activates the mesolimbic DA pathway [45], while Wang et al. provided electrophysiological evidence linking improved inhibitory control from chronic aerobic exercise to reduced craving [25]. Meanwhile, the exercise-regulated protein kinase A inhibitor (PKI) suppresses the D1 receptor–PKA–CREB signalling axis, blocks the reconsolidation of Meth use disorder-related memories, and reduces cue-induced relapse behaviours [15]. Exercise promotes synaptic regeneration by upregulating synaptophysin (SNP) expression, reversing Meth-induced damage to DA and serotonergic nerve terminals. This repair mechanism alleviates withdrawal-related negative emotions like anxiety and depression [46]. Furthermore, exercise exerts comprehensive neuroprotection by bidirectionally regulating Glu/GABA homeostasis to rebalance synaptic excitation-inhibition, optimising cerebral blood flow and metabolism, and activating neurotrophic and antioxidant pathways [16]. The overall research framework integrating these multidimensional mechanisms is presented in Figure 3.

In summary, exercise offers a multi-dimensional integrated therapeutic strategy that synergistically targets reward craving, addiction memory, and emotional–cognitive dysfunction, offering a non-invasive, low-risk, and sustained intervention for Meth abstinence. A critical moderating factor is the baseline neurodamage level; efficacy is dose-dependent, wherein severe damage from chronic high-dose Meth use necessitates prolonged moderate-intensity exercise, whereas neurochemical imbalance from short-term use responds more rapidly to shorter protocols.

### 4.2. Effect of Exercise Type on Craving in Meth Use Disorder

Drug craving is defined as an irresistible desire for previously experienced psychoactive substances among drug abusers [47]. Psychological craving in individuals with Meth use disorder is a key factor leading to drug-seeking behaviour and eventual relapse after abstinence [11]. Therefore, reducing Meth craving is a critical component of Meth use disorder rehabilitation. The following sections explore how exercise improves Meth craving through aerobic exercise, resistance training, combined aerobic and resistance training, and mind–body exercises.

#### 4.2.1. Aerobic Exercise Reduces Craving in Meth Use Disorder

Recent studies have demonstrated that regular aerobic exercise can reverse these pathological processes through a multi-target intervention mechanism. Saanijoki et al. [45] showed that moderate aerobic exercise activates the mesolimbic DA pathway, promoting endogenous DA release to compensate for neurotransmitter deficits during withdrawal, thereby alleviating negative affect and physiological discomfort while significantly attenuating drug craving. Clinical randomised controlled trials have demonstrated that 12 weeks of moderate-intensity aerobic exercise, 3 times per week for 30 min each time, can enhance the behavioural inhibition ability of Meth abusers, and the intervention effect increases dose-dependently with the prolongation of the cycle [25]. Thus, moderate-intensity aerobic exercise (3–5 sessions/week, ≥30 min/session, for 2–3 months) represents a universal threshold for ameliorating Meth craving and withdrawal symptoms. Chen et al. [48] demonstrated that high-intensity exercise exerted more pronounced inhibitory effects on craving levels in male abstainers. This suggests that optimal exercise intensity may be population-dependent. In contrast, Zhao et al. [23] specifically demonstrated the efficacy of chronic, moderate-intensity aerobic exercise in reducing attentional bias among female Meth users, highlighting the importance of considering gender-specific factors in prescription design.

For the intervention of acute cravings, studies on human subjects provide direct behavioural evidence. For instance, Li et al. [49] found that a single 10-minute virtual reality competitive cycling session rapidly activates prefrontal inhibitory control functions, suggesting that aerobic exercise can be an effective strategy for managing acute cravings. At the mechanistic level, animal model studies provide crucial insights into the molecular and cellular mechanisms underlying disease. For example, voluntary wheel running in Meth-exposed mice upregulates hippocampal vascular endothelial growth factor and brain-derived neurotrophic factor (BDNF), thereby promoting angiogenesis and synaptic remodelling [50]. These findings provide a plausible mechanistic hypothesis for how exercise may reverse Meth-induced oxidative stress and cognitive deficits; however, the exact role of these specific molecular pathways in human addicts requires further validation through studies combining neuroimaging and blood biomarkers. However, the efficacy of aerobic exercise is not uniform across all individuals. Factors such as baseline fitness, severity of cognitive impairment, and genetic predispositions (e.g., BDNF Val66Met polymorphism) may moderate the response. Future personalised approaches should consider these variables to identify individuals who are likely to respond.

#### 4.2.2. Effects of Resistance Training on Craving in Meth Use Disorder

In terms of emotional improvement, research indicates that acute resistance training not only modulates emotional states in alcohol and tobacco-dependent individuals but also significantly enhances positive emotional experiences in those with Meth use disorder [51]. More importantly, resistance training exerts similar effects in alleviating anxiety symptoms and enhancing pleasant feelings in healthy populations [52], suggesting that the positive affective response to resistance training may be a fundamental psychophysiological reaction, not specific to a particular substance dependence. This strengthens the rationale for employing resistance training as an emotional intervention for individuals with Meth use disorder.

In terms of cognitive function, resistance training enhances inhibitory control and selective attention—two cognitive abilities closely related to the regulation of attentional bias [53,54]. Efficacy is intensity-dependent: moderate-intensity (55–60% 1 RM) training outperforms low-intensity protocols in reducing cravings and improving mood [22]. Additionally, 12 weeks of such training enhances functional connectivity in brain regions related to emotional control and cognition, further strengthening craving resistance [21].

In summary, resistance training provides multi-faceted support for Meth use disorder rehabilitation by concurrently improving emotional state, enhancing cognitive function, and inducing beneficial neuroplasticity, with moderate-intensity training demonstrating particular promise.

#### 4.2.3. Comparison of Aerobic and Resistance Training Effects on Improving Meth Craving Levels

A comparative synthesis reveals complementary benefits of aerobic and resistance training, mediated by distinct mechanisms that support sequential or combined protocols. Aerobic exercise has been shown to demonstrate superior efficacy in rapidly reducing immediate cravings, likely through modulation of the mesolimbic dopamine system [45,47]. In contrast, resistance training more effectively enhances emotional stability, impulse control, and long-term craving reduction, potentially through its influence on the amygdala–striatal circuit and serotonin levels [21,54]. This mechanistic distinction is clear: aerobic exercise alleviates reward deficiency by activating the dopaminergic system, while resistance training improves mood stability by modulating serotonergic function [55]—a benefit evident even after a single session [27].

The logical synergy between these modalities is empirically supported. For instance, Guo et al. [56] reported that combined training significantly reduced anxiety and craving in male opioid-dependent patients, a finding consistent with Rawson et al.’s [19] RCT demonstrating sustained relapse prevention benefits for Meth use disorder. Furthermore, mind–body exercises like Chan-Chuang, which integrate aerobic components, exemplify this synergy by regulating the autonomic nervous system to maintain long-term emotional stability and abstinence [26]. Combined interventions thus synergise the rapid anti-craving effects of aerobic exercise with the executive control enhancement from resistance training. Consequently, the evidence not only distinguishes the unique advantages of each modality but also provides a mechanistic rationale for a sequential intervention paradigm: employing aerobic exercise to address acute reward deficiency during early abstinence, followed by incorporating resistance training to consolidate long-term emotional and executive control. Their complementary profiles are directly compared in Table 2.

#### 4.2.4. Effects of Mind–Body Exercises on Craving in Meth Use Disorder

Mind–body exercise, centred on ‘mind–body co-ordinated regulation,’ integrates structured physical activity, attention regulation, and breathing rhythm coordination to exert therapeutic effects [57], with core forms including mindful yoga, qigong, and tai chi. Beyond enhancing flexibility and balance through physical training (e.g., yoga asana stretches, tai chi centre-of-gravity shifts), these practices strengthen attention regulation and mind–body coordination through integrated physical–mental engagement [58].

Mechanistically, the mindfulness-based cognitive therapy component guides individuals with Meth use disorder to non-judgmentally perceive drug cravings, fostering cognitive acceptance of negative emotions and impulses. This breaks the “craving-impulse-relapse” cycle and mitigates craving levels [59]. Empirically, Ding et al.’s [17] randomised controlled trial (40 female participants) confirmed that a combined yoga-meditation-physical training programme reduced drug-seeking behaviours and long-term relapse risk, while Petker et al. [60] highlighted yoga’s role in improving self-efficacy, impulse control, and mindfulness as an adjunctive treatment. Systematic reviews and meta-analyses further validate that traditional mind–body exercises (e.g., tai chi, qigong) alleviate depressive/anxiety symptoms, enhance quality of life and sleep, and reduce drug dependence and relapse risk among drug users [61]; notably, a tai chi-focused study on female Meth users specifically demonstrated reduced craving frequency and intensity [62].

Collectively, these multi-angle studies establish mind–body exercise as a robust adjunctive rehabilitation intervention for Meth use disorder, supported by mechanistic insights and empirical evidence spanning individual trials to synthetic analyses.

### 4.3. Exercise Intervention Improves Psychosocial Functioning in Meth Use Disorder

In addition to alleviating Meth cravings, scientific exercise training reduces psychological dependence on Meth. It enhances abstinence willpower in individuals with substance use disorder by improving self-efficacy and social support networks, thereby facilitating the establishment of a positive lifestyle [63]. This mechanism is reflected in specific research findings. For instance, Cui et al. [61] demonstrated that tai chi exercise significantly alleviates withdrawal-induced anxiety and negative emotions associated with depression by modulating the balance of the autonomic nervous system and promoting endorphin release, while concurrently enhancing self-confidence and stress resilience. Research focusing on specific populations has shown that group dance therapy for female individuals in recovery accelerates psychosocial functional recovery by activating the mirror neuron system and enhancing empathetic behaviours, thereby improving emotional regulation capacity and social skills [23]. Furthermore, at the neurofunctional level, Gao et al. [64] found that after high-intensity interval training, male patients with Meth use disorder exhibited significantly enhanced oxygenation levels in the prefrontal cortex and functional connectivity between the default mode network and synaptic network, which improved cognitive function and emotion regulation efficiency. Thus, appropriate exercise facilitates the formation of a positive lifestyle attitude in individuals with use disorder, establishing dual neurobiological and psychological foundations for long-term rehabilitation.

### 4.4. Exercise Enhances Physical Health in Meth Use Disorder Patients

Chronic Meth abuse induces multi-system dysfunction, and scientific evidence indicates that regular exercise can effectively promote functional recovery across multiple physiological systems in abstinent individuals. At the neurological level, Zhu et al. [65] employed moderate-intensity aerobic exercise for 60 min, five times per week, to modulate vascular endothelial homeostasis, reduce blood–brain barrier permeability, and attenuate neuroinflammation and oxidative damage. Regarding metabolic homeostasis, exercise also activates the AMPK-PPARα signalling pathway, reduces low-density lipoprotein (LDL) levels, and effectively inhibits atherosclerotic pathology [66]. Clinical intervention studies further substantiate the feasibility and comprehensive benefits of exercise protocols. For instance, Rawson et al. [24] and Dolezal et al. [67] demonstrated that an 8-week progressive combined aerobic-resistance training protocol (initial phase: 30 min/session at 60% HRmax; advanced phase: 60 min/session at 80% HRmax) significantly ameliorated Meth withdrawal-induced somatic anxiety symptoms and reduced relapse rates. Collectively, this evidence suggests that exercise, through the synergy of motor, neurological, and metabolic functions, provides a systematic solution for functional rehabilitation in Meth use disorder.

## 5. Exercise Prescription for Meth Use Disorder Formulation

As discussed earlier, exercise interventions have been shown to facilitate rehabilitation in Meth use disorder effectively. When designing exercise rehabilitation programs, in addition to adhering to the fundamental FITT-VP principles, careful consideration must be given to intervention timing, individual health status, and exercise preferences.

### 5.1. Timing of Exercise Interventions for Meth Use Disorder

Current evidence indicates that exercise interventions exert stage-dependent neurobehavioral modulation effects on Meth abstinence, necessitating the development of individualised protocols tailored to the progression of withdrawal [68]. During the acute withdrawal phase (0–3 months), exercise significantly suppresses drug craving by activating alternative reward pathways [69]. Roessler et al. [70] demonstrated that nearly half of individuals with use disorder who received exercise interventions exhibited reduced drug use during the initial phase, suggesting that exercise exerts an immediate inhibitory effect on acute withdrawal behaviour. Longitudinal studies of stratified effects during the withdrawal maintenance period showed that the long-term impact of exercise was significant in mild use disorder, with drug intake reduced by approximately 75% 1–6 months post-intervention compared to the control group. In contrast, in individuals with severe Meth use disorder, a single exercise intervention had a limited effect on reversing drug cravings due to severe neurological damage and substantial behavioural consolidation [19]. Therefore, the timing of exercise intervention must align with the neuroplasticity-sensitive windows and behavioural remodelling nodes during the withdrawal process. This alignment facilitates the construction of an exercise-reward neural substitution pathway, provides a neurobehavioral basis for formulating appropriate exercise prescriptions, and ultimately achieves a closed-loop intervention from physiological detoxification to psychological rehabilitation.

### 5.2. Development of Exercise Prescriptions for People in Different Stages of Meth Use Disorder

Based on drug rehabilitation practical experience and gradually emerging medical theoretical consensus, the abstinence process of drug abusers is divided into three stages: physiological detoxification, rehabilitation consolidation, and social reintegration coaching. Combined with the Consensus on Exercise Prescription for Drug Abusers [71], exercise prescriptions have been developed for these three stages in the Meth use disorder population.

#### 5.2.1. Exercise Prescription for the Physiological Detoxification Stage in Meth

Individuals in the physiological detoxification phase typically experience intense withdrawal symptoms, including severe anxiety and pain. During this phase, the primary goal of exercise interventions for individuals with Meth use disorder is to alleviate withdrawal-induced pain and reduce early abstinence-related physiological and psychological discomfort. This facilitates a smooth transition through the acute detoxification period while progressively restoring physical functions and cultivating sustainable exercise habits. The exercise prescription should prioritise adaptive training, with aerobic exercise as the primary modality to implement low-energy-expenditure and low-intensity physical rehabilitation protocols. Recommended activities include slow walking and health-preserving qigong, administered 3 times weekly for 10–30 min per session at approximately 40% of maximum heart rate [72]. This protocol enhances circulatory function, promotes the release of endorphins, and relieves withdrawal symptoms—particularly suitable for high-dose chronic users to avoid exacerbating residual neurophysiological stress.

#### 5.2.2. Exercise Prescription for Rehabilitation Consolidation Phase in Meth Recovery

During the rehabilitation consolidation phase, while physiological dependence in individuals with Meth use disorder is largely controlled, psychological and social factors may still significantly hinder recovery progress. At this stage, the exercise prescription should implement targeted, high-energy-expenditure, and moderate-intensity physical rehabilitation training, combining aerobic exercise (primary) with resistance training (supplementary). This approach is grounded in clinical evidence. The feasibility and benefits of such combined protocols have been demonstrated in studies by Rawson et al. [66] and Dolezal et al. [67]. Furthermore, the effectiveness of integrating mind–body practices, such as Chan-Chuang, with resistance training during this phase was specifically demonstrated by Li et al. [26] in a study of Chinese males.

Aerobic component: Running, ball games, and dance-based activities at 60–70% of maximum heart rate, administered 3 times/week for 30 min/session. Resistance component: Machine-based and bodyweight resistance training at 60–70% 1 RM intensity, performed 3 times/week (1–4 sets/session, 10–15 repetitions/set) with 3 min inter-set rest periods and 15–30 min/session duration. During this phase, the enrichment and intensification of training content help enhance the physical function of individuals with Meth use disorder, boost their self-confidence, and improve self-efficacy [60], thereby laying a foundation for subsequent social reintegration. For low-dose short-term users, intensity can be moderately increased (e.g., aerobic exercise at 65–70% maximum heart rate) to accelerate neurofunctional recovery.

#### 5.2.3. Exercise Prescription for Social Reintegration Coaching in Meth Recovery

At the critical stage of social reintegration for individuals with Meth use disorder, it is necessary to establish a supportive sports rehabilitation environment through the collaboration of the families, communities, and medical institutions. This collaboration aims to consolidate the effects of previous interventions and prevent relapse into drug abuse. Family members can strengthen the social connection and family responsibility of the person in withdrawal by participating together in a customised exercise programme [73]. Communities should develop specialised exercise rehabilitation programmes for drug abstinence, incorporating traditional Chinese fitness regimens (e.g., health-preserving qigong and Baduanjin) to create low-threshold, destigmatised exercise environments. Concurrently, peer-supported exercise groups should be organised to address social relationship deficits through mutual motivation among individuals with Meth use disorder [74]. Evidence suggests that gender-specific exercise preferences exist, with males favouring high-intensity group activities and females tending toward flexibility training [75]. Medical teams should develop gender-differentiated exercise prescriptions based on neurofunctional assessments and movement preferences of use disorder to optimise therapeutic outcomes, e.g., prolonged moderate-intensity training for high-dose chronic users to consolidate neuroplasticity gains, and community-based variable-intensity exercise for low-dose users to reinforce behavioural adaptation. A social support network-driven ecological rehabilitation system can facilitate lifestyle reconstruction, enabling a seamless transition from physiological abstinence to social reintegration and functional recovery.

### 5.3. The Role of Age and Gender in Exercise Interventions

Developing individualised exercise rehabilitation programmes requires careful consideration of age and gender factors, as these variables significantly influence both the clinical presentation of Meth use disorder and the response to exercise interventions. For adolescents, characterised by high impulsivity and novelty-seeking, prescriptions should prioritise short-duration, gamified training to enhance engagement, consistent with evidence-based approaches for this demographic [76]. In elderly patients, age-related declines in neuroplasticity and elevated cardiovascular risks necessitate low-intensity, community-based activities [77].

Gender differences further dictate prescription design. Males typically respond better to high-intensity group sports (e.g., ball games) and resistance training, which align with clinical findings on craving reduction [22] and surveyed preferences for activities that satisfy social dominance needs [75]. Conversely, prescriptions for females should emphasise mind–body integration and endocrine regulation. This is particularly important for perimenopausal and postmenopausal women, for whom tailored protocols, such as moderate-intensity aerobic exercise [78] and mind–body practices (e.g., Tai Chi), have demonstrated efficacy in managing cravings and improving cardiovascular health [61]. In summary, stratifying exercise prescriptions based on age, gender, and physiological stages is fundamental to optimising outcomes across diverse populations. These principles are translated into specific clinical protocols, as outlined in Table 3.

### 5.4. Impact of the Rehabilitation Environment on the Implementation of Exercise Intervention

After developing age- and gender-specific phased exercise prescriptions (Table 3), the support of the rehabilitation environment becomes crucial to ensuring their effective implementation. The rehabilitation environment, as a tangible manifestation of socioeconomic status and social context, directly determines the efficacy and sustainability of exercise interventions. Its core strategy lies not in the simple ‘removal’ of high-risk environments, but in constructing a sequential ecological transition plan involving ‘controlled protection-skill acquisition-graded exposure.’ During acute withdrawal, physical isolation in inpatient or closed-door facilities creates a secure window for neural repair during intensive exercise interventions [24]. Concurrently, group exercise itself fosters a favourable microenvironment, replacing drug-oriented social patterns with healthy peer connections [74]. In the later rehabilitation phase, low-threshold community exercise programmes (e.g., running groups, fitness classes) serve as low-risk ‘behavioural testing grounds,’ enabling patients to generalise and consolidate acquired emotional regulation and impulse control skills within supportive environments [27,75]. This ‘institutional grounding-community integration’ environmental adaptation strategy addresses the differentiated needs of patients from diverse socioeconomic backgrounds, while highlighting the dual attributes of exercise intervention: physiological restoration and social construction. It lays the practical foundation for the subsequent development of standardised assessment systems encompassing ‘social functional recovery’ and personalised treatment planning [73,74].

### 5.5. Evaluation Criteria for Exercise Interventions in Promoting Rehabilitation of Meth Use Disorder

**Figure 4:**
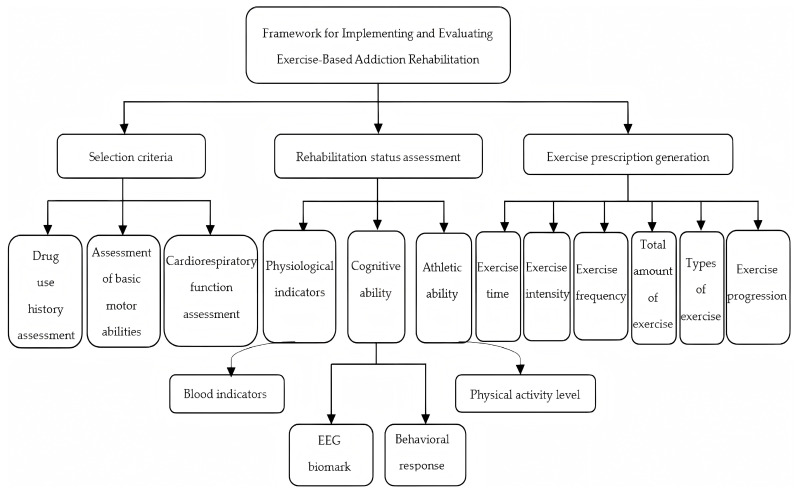
Schematic diagram of exercise-based drug rehabilitation implementation and evaluation criteria.

To realise scientific exercise rehabilitation for Meth use disorder, establishing a standardised assessment system is imperative. China has thus developed and implemented the Rehabilitation Assessment Guidelines for Physical Exercise in Individuals Under Compulsory Isolation for Drug Detoxification [79], which integrates the Exercise Prescription theoretical framework [80] and specialised assessment software [81] to form a comprehensive Meth abstinence rehabilitation evaluation standard. This standard is operationalised through the Framework for Implementing and Evaluating Exercise-Based Addiction Rehabilitation, which outlines a three-tiered progressive intervention system (Figure 4):

(1) A tri-level screening protocol (incorporating drug use history, basic motor capacity, and cardiopulmonary function) using standardised questionnaires and medical imaging to exclude high-risk individuals;
(2) A physiological-cognitive-motor triaxial assessment model integrating blood biomarkers, EEG spectral features, and behavioural data for quantitative evaluation of neurofunctional impairment in drug use disorder;
(3) A personalised exercise prescription system dynamically modulated by FITT-VP principles (exercise intensity: 40–70% VO_2_ max; progression rhythm: ≤10% weekly load increment).

During implementation, wearable devices and functional near-infrared spectroscopy enabled real-time matching of exercise load with cerebral hemodynamic responses. Periodic reassessment-driven optimisation of prescription parameters established a long-term adaptation mechanism, spanning structured in-hospital training to community-based self-exercise regimens. This system achieves end-to-end standardisation through the screening–assessment–prescription workflow, deeply integrating exercise science into Meth use disorder clinical practice. Biochemically, it modulates neuroplasticity and restores metabolic homeostasis; behaviourally, it reinforces exercise routines; socially, it facilitates the ecological transfer of healthy behaviours.

In summary, the Exercise-Based Drug Rehabilitation Implementation and Evaluation Standards framework provides a preliminary, systematic solution that unifies scientific rigour and practical applicability through multidimensional integration. As a novel theoretical framework, its validity and reliability require further validation through rigorous empirical research, including large-sample randomised controlled trials to demonstrate superiority over traditional methods and quantitative analysis of each evaluation indicator’s predictive validity. Nevertheless, the framework provides the global addiction treatment field with a clear, testable pathway for evidence-based research, offering substantial theoretical value and practical guidance.

## 6. Summary and Discussion

### 6.1. Contributions

In conclusion, this review establishes exercise as a precise, mechanism-informed adjunct treatment for Methamphetamine (Meth) use disorder by proposing an integrative ‘Exercise Modality–Neural Target–Rehabilitation Stage’ framework. It synthesises evidence demonstrating that distinct exercise modalities (aerobic, resistance, and mind–body) differentially and complementarily target the specific neurobiological deficits underlying the disorder [17,18,19]. A key mechanistic insight is the role of exercise in restoring the imbalance in Glu/GABA neurotransmitter systems, a core pathology of Meth addiction.

The primary theoretical contribution is the integration of disparate evidence into a coherent model, shifting the paradigm of exercise from a general wellness aid to a targeted neurotherapeutic strategy. This provides a principled basis for personalisation. For clinical practice, this review translates mechanistic insights into a phased intervention approach: (1) prioritising aerobic exercise in the acute withdrawal phase to alleviate cravings via dopamine pathway activation [25,45]; (2) employing combined aerobic-resistance training during rehabilitation consolidation to address both reward deficiency and emotional dysregulation [66,67]; and (3) introducing community-based mind–body exercises to facilitate long-term social reintegration [73,75]. Future research should prioritise dose–response studies and explore the integration of exercise with neuromodulation therapies to further standardize and optimize these interventions.

### 6.2. Discussion

A critical appraisal reveals significant methodological heterogeneity in the evidence base, evident in variations in exercise protocols (e.g., high-intensity [48] vs. chronic moderate-intensity aerobic exercise [23,49]) and population characteristics (e.g., gender-specific responses [23,48]). Despite these disparities, a convergent pattern emerges: different exercise modalities work in complementary ways to reverse Methamphetamine-induced Glu/GABAimbalance. This overarching neurobiological mechanism provides a robust foundation for our model. Consequently, apparent efficacy contradictions across studies likely reflect methodological and population disparities rather than true inefficacy of the intervention, complicating the identification of universal principles. Several limitations must be acknowledged. As a narrative review, our qualitative synthesis may be subject to selection bias despite efforts to be comprehensive. Furthermore, the proposed mechanistic pathways, while plausible, often rely on indirect evidence from animal studies or correlational human data.

These challenges are multifaceted. First, the causal inference regarding long-term neuroplasticity, particularly in key systems such as the Glu/GABA loop, remains speculative due to a paucity of longitudinal studies. Most existing evidence, such as the neuroimaging changes observed by Gao et al. [64], captures correlational rather than causal relationships over time. Furthermore, the synergistic potential of exercise with other therapies and the integration mechanisms of social support networks are underexplored, a situation exacerbated by the lack of quantitative adherence metrics and the unclear long-term efficacy of social interventions. Critically, the absence of established strategies for adapting prescriptions to diverse socio-cultural backgrounds severely limits the generalizability and real-world impact of interventions.

The current understanding of the underlying mechanisms, while compelling, remains hypothetical and is constrained by several methodological limitations. A systematic review of exercise interventions for methamphetamine dependence identified only three eligible studies, with an average PEDro score of 6.66, indicating moderate methodological rigour [82]. Furthermore, the global evidence base consists of only a handful of small-scale RCTs, which are often underpowered (e.g., one recent RCT included 120 participants across four subgroups) [83]. A fundamental conceptual challenge is the failure to differentiate acute, transient effects from chronic, adaptive neuroplasticity. This conflation of immediate psychological benefits with long-term therapeutic outcomes obscures mechanistic insights. As emphasised by Etnier et al. [84], clarifying this distinction—such as the differential temporal dynamics between exercise-induced BDNF changes and cognitive improvements—is crucial. Addressing these limitations necessitates more standardised protocols and high-quality RCTs.

### 6.3. Future Outlook

Building on the ‘Exercise Modality–Neural Target–Rehabilitation Stage’ framework proposed in this review, we conclude that exercise is not merely an adjunctive wellness activity but a targeted therapeutic strategy with multi-level benefits: at the neurobiological level, it restores neurotransmitter homeostasis; at the clinical level, it provides a non-pharmacological alternative for craving management; and at the social level, it supports long-term recovery through ecological integration. To translate these findings into practice, we recommend: (1) Incorporating exercise assessment into routine clinical evaluation for Meth use disorder; (2) Developing professional training programmes for exercise rehabilitation specialists; and (3) Establishing community-based exercise facilities specifically designed for substance use recovery. These recommendations are supported by existing evidence from both clinical trials and implementation studies [14,19,79].

## Figures and Tables

**Table 1:** Selected Core Literature Relevant to This Study.

Research (Year)	Evidence Levels and Design	Research Subjects and Core Interventions	Key Performance Indicators	Core Contributions to the Theoretical Model
Alessi et al. (2020) [14]	RCT	Outpatients with Substance Use Disorders: Intensive Exercise (3 times/week, 12 weeks)	Relapse rate, treatment adherence	Provide Level I evidence that long-term regular exercise reduces relapse as a hard endpoint during community rehabilitation
Ding et al. (2023) [17]	Controlled trial	Female Meth users: Different types of exercise (e.g., aerobic/yoga, 2–3 times per week × 8 weeks)	Emotional state, level of drug craving	Exercise Regulates Neurotransmitters to Improve Mood and Cravings. Group-matched control bias; generaliza-ble to similar female postmenopausal populations
Jayanthi et al. (2021) [20]	Literature Review	Integrate existing evidence to elucidate the neurotoxic mechanisms of Meth	Mechanism Analysis	Provide the theoretical basis for Meth-induced neurological damage and identify neuroprotective targets for exercise intervention
Li et al. (2021) [21]	RCT	Meth use disorder patients: moderate-intensity resistance training (12 weeks)	Brain functional connectivity metrics (fMRI detection)	Provide mechanistic evidence that resistance training improves functional connectivity within brain networks
Peng et al. (2021) [22]	Dose–response study	Meth use disorder patients; different intensities of resistance training (8–12 weeks)	Psychological craving score, response to triggers	Revealing a dose-dependent relationship between resistance training intensity and craving reduction
Zhao et al. (2024) [23]	RCT	Female Meth addicts, chronic aerobic exercise at moderate intensity (3 times/week × 12 weeks)	Attention bias	Evidence suggests that chronic exercise enhances cognitive control and reduces attentional bias toward drug cues
Rawson et al. (2015) [24]	RCT	Meth-dependent individuals after hospitalisation; Combined aerobic and strength training (12 weeks)	Relapse rate, frequency of use	Evidence confirms that combined exercise offers sustained benefits in consolidating long-term abstinence and preventing relapse
Wang et al. (2017) [25]	RCT	Meth dependence in individuals: aerobic exercise (3 times per week, 12 weeks)	Rating craving, inhibitory control, ERP	Provides electrophysiological evidence linking recent improvements in motor and inhibitory control functions to reduced craving
Li et al. (2023) [26]	RCT	Chinese male Meth users; Chan-Chuang + resistance training	Craving intensity, withdrawal symptoms, and treatment adherence	Demonstrate the comprehensive rehabilitative benefits of mind–body integrated exercise patterns among the Chinese population.
Jin et al. (2025) [27]	Intervention-controlled study	Meth use disorder patients: acute aerobic exercise vs. resistance training of equal intensity	Craving, Emotion, Cognitive Function	Directly comparing the acute effects of different exercise types provides a basis for personalised prescriptions

Note: This table presents a condensed version focusing on the most significant findings. It outlines the core evidence base (10 items total) supporting the integrated model of ‘exercise pattern–neural target–rehabilitation phase’.

**Table 2:** Comparative analysis of exercise modalities in Meth use disorder rehabilitation.

Parameters	Aerobic Exercise	Resistance Training	Mind–Body Exercise
Primary Neural Target	Prefrontal–striatal circuit (cognitive control)	Amygdala–striatal circuit (emotional regulation)	Autonomic Nervous System/Insula (interoception)
Primary Mechanism	Upregulates BDNF, enhances dopamine D1 receptor function	Modulates dopamine/serotonin balance, increases GABAergic tone	Increases heart rate variability, reduces cortisol enhances mindfulness
Key Symptom Target	Cognitive impairment, attentional bias	Emotional dysregulation, impulse control	Craving, anxiety, stress reactivity
Optimal Intervention Stage	Early abstinence (cognitive rehab), Maintenance	Rehabilitation consolidation (emotional stability)	Across all stages (esp. stress management and relapse prevention)
Typical Protocol	30–50 min, 60–75% HRmax, 3–5×/week	60–70% 1 RM, 3 sets of 8–12 reps, 2–3×/week	30–60 min, low-moderate intensity, 3–7×/week

**Table 3:** Summary of Stage-Specific Exercise Intervention Protocols for Meth Use Disorder.

Parameters	Physiological Detoxification Stage	Rehabilitation Consolidation Phase	Social Reintegration Coaching
Primary Goals	Alleviate withdrawal symptoms, reduce acute cravings, and establish an exercise routine.	Improve emotional stability, enhance cognitive function, and prevent relapse	Rebuild healthy lifestyles, promote social integration, and consolidate long-term abstinence.
Recommended Modalities	Primarily aerobic exercise	Combining aerobic and resistance training	Physical and Mental Exercise and Community Group Activities
Exercise Intensity	Low intensity (approximately 40% of maximum heart rate)	Moderate intensity as the primary focus (Cardio: 60–70% of maximum heart rate; Resistance training: 60–70% of 1 RM)	Moderate intensity, emphasising enjoyment and sustainability
Age and Gender Considerations	Adolescents: Focus on short-duration, highly engaging gamified exercise to prevent resistance. Perimenopausal women: Prioritise bone health and incorporate low-impact resistance training.	Adult males: Team-based competitive activities can be introduced to satisfy social dominance needs; Females: Strengthen practices like yoga and tai chi that simultaneously improve emotional well-being and body image.	**Women:** Recommended to prioritise group aerobics. **Men:** Focus on group ball sports. **Older adults:** Engage primarily in low-impact activities like walking or tai chi to enhance well-being.
Frequency & Duration	3 times per week, 10–30 min per session	3–5 times per week, 30–60 min per session	≥3 times per week, 30–60 min per session, encouraged to integrate into daily life
Key Neural Targets	Activate the mesolimbic dopamine pathway to compensate for reward deficiency; promote endorphin release.	Regulate the amygdala–striatal circuit to improve emotional control, and enhance prefrontal cortex function to strengthen inhibitory control.	Balance the autonomic nervous system to manage stress and cravings; Promote brain health through social engagement.
Implementation Environment	Closed institution	Semi-open institutions/Community transition zones	Community + Family

## Data Availability

Data sharing is not applicable to this article, as no datasets were generated or analysed during the current study.

## Supplementary Materials

The following supporting information can be downloaded at: https://www.mdpi.com/article/10.3390/brainsci15121339/s1, Table S1: Selected Core Literature Related to This Study.

## Abbreviations

The following abbreviations are used in this manuscript:

Meth	Methamphetamine
VTA	ventral tegmental area
PFC	prefrontal cortex
GABA	gamma-aminobutyric acid
PKI	Protein Kinase Inhibitor Peptide
DA	dopamine
NAc	nucleus accumbens
HIPP	hippocampus
TH	tyrosine hydroxylase
SNP	Synaptophysin
Glu	Glutamatergic
AMY	amygdala
DAT	dopamine transporter
GPCR	G protein-coupled receptor
