# Supplementary material for: Exercise as a Promising Adjunct Treatment for Methamphetamine Addiction: Advances in Understanding Neuroplasticity and Clinical Applications
Source: Brain Sci. 2025 Dec 16;15(12):1339. doi: 10.3390/brainsci15121339 (PMC12730288; doi:10.3390/brainsci15121339)
Supplement: Supplementary file 1 [file brainsci-15-01339-s001.zip › brainsci-4010305-supplementary.pdf]

This document serves as supplementary material to the main text 'Table 1: Selected Core Literature Relevant to This Study,' providing a detailed review of 15 relevant publications within the 10 streamlined references listed in Table 1 of the main text.

Supplementary Table S1. Selected Core Literature Related to This Study

Researcher	Research Methods	Research Group	Intervention measures	Evaluation Method	Mechanism of Action	Research Quality
Alessi et al. (2020)	RCT	Outpatients with Substance Use Disorders	Intensive exercise intervention; 3 times/week×12 weeks	Relapse rate, treatment adherence	Exercise Improves Physical and Mental Health to Aid in Drug Rehabilitation	Randomised group assignment reduces bias; generalizable to similar categories.
Ding et al. (2023)	Controlled trial	Female Meth users	Different types of exercise (e.g., aerobic/yoga, 2-3 times per week ×8 weeks)	Emotional state, level of drug craving	Exercise Regulates Neurotransmitters to Improve Mood and Cravings	Group-matched control bias; generalizable to similar female postmenopausal populations
RAWSON et al. (2015)	RCT	Meth addicts (following inpatient treatment)	Exercise intervention (such as regular aerobic exercise + strength training, lasting 12 weeks)	Relapse rate, frequency of use	Exercise reduces addiction-related neural cravings	Follow-up tracking controls for bias can be extended to post-hospitalisation addiction rehabilitation populations.
JAYANTHI et al. (2021)	Literature Review	Integrate existing research	Mechanism Analysis	Neurotoxic Effects of Meth	Inducing neural damage through oxidative stress, neuroinflammation, and other mechanisms	Systematically review multiple research studies applicable to neurotoxicity mechanisms.
Chen et al. (2019)	Controlled trial	Meth Dependent Individuals	Acute aerobic exercise of varying intensities (e.g., low/moderate/high intensity, single-session	Drug Craving Score, Cognitive Function Indicators	Acute aerobic exercise influences craving levels through cognitive modulation.	Group-balanced baseline; generalizable to the abstinence period.

			intervention)			
Li et al. (2022)	Controlled trial	Meth Dependent Individuals	Acute exercise intervention (single session) + drug cue exposure	Drug craving intensity, cortical hemodynamic parameters	Acute motor regulation of cortical function reduction induces cue-induced craving	Establish a cue exposure control, extendable to studies on cue-reactivity interventions in dependent individuals.
Li et al. (2021)	RCT	Individuals with Methylamphetamine Use Disorder	Moderate-intensity resistance training (e.g., 2–3 times/week × 12 weeks, progressive overload)	Brain functional connectivity metrics (fMRI detection)	Resistance training improves brain functional connectivity by modulating neural pathways.	Group-matched baseline; generalizable to rehabilitation intervention studies in similar patient populations
Peng et al. (2021)	Dose-Response Interventional Controlled Study	Individuals with Methylamphetamine Use Disorder	Resistance training at varying intensities (e.g., low/moderate/high load, 2-3 times per week × 8-12 weeks)	Psychological craving score, response level to craving-inducing factors (cues/stress, etc.)	Resistance training modulates neurotransmitters and emotional states, exerting a dose-dependent influence on cravings.	Group-balancing dose factor; applicable to studies optimising intervention doses for craving in dependent individuals
ZHAO et al. (2024)	RCT	Female Meth addicts	Aerobic exercise + resistance training (regular intervention, e.g., 2–3 times/week× 8–12 weeks)	Attention Bias (Drug-Related Cues)	Chronic exercise modulates cognitive control and attentional bias toward weakened cues.	Group control baseline; generalizable to other female addicts
Somkuwar et al. (2015)	Intervention Effect Evaluation Research	Meth Addiction-Like Behaviour Model/ Addiction Population	Aerobic exercise + resistance training (regular intervention, e.g., 2–3 times/week× 8–12 weeks)	Relapse rate, craving intensity, conditioned preference	Regulating reward pathways through exercise and improving emotional regulation to mitigate addictive beh	Establish controls and replicate validation; extend to clinical research on addiction-related exercise interventions.

					aviours	
Arazi et al. (2017)	RCT	Male Meth addicts	Aerobic exercise + resistance training (regular intervention, e.g., 2–3 times/week×8–12 weeks)	Neurotransmitter levels (e.g., dopamine, serotonin), cardiovascular response indicators (heart rate, blood pressure)	Exercise improves cardiovascular function by regulating neurotransmitter balance.	Group-matched baseline characteristics; generalizable to studies on cardiovascular and neurotransmitter-related aspects of exercise rehabilitation in men with addiction
Wang et al. (2017)	RCT + Event-Related Potential Study	Meth Dependent Individuals	Aerobic exercise training (regular intervention, e.g., 3 times/week×12 weeks, moderate intensity)	Drug Craving Score, Inhibitory Control Ability, Event-Related Potential Measures (e.g., N2/P3 Amplitude)	Aerobic exercise reduces drug cravings in people with an addiction by improving inhibitory control functions.	Randomised grouping balanced confounding factors; combined with electrophysiological indicators, the evidence is highly credible and can be extended to studies on cognitive function interventions in the motor rehabilitation of individuals with dependency.
Gao et al. (2022)	RCT	Male Meth addicts	Moderate-to-high intensity acute aerobic exercise (single intervention)	Oxygenation Levels in the Prefrontal Cortex (Near-Infrared Spectroscopy)	Acute aerobic exercise influences prefrontal cortex oxygenation by regulating blood flow perfusion.	Group-balanced exercise intensity variables; generalizable to male-dependent exercise-related cerebral oxygenation studies
Li et al. (2023)	RCT	Male Meth users in China	Chan-Chuang+Resistance Training	Drug craving intensity, withdrawal symptom scores, and treatment adherence	The Chan-Chuang exercise, combined with resistance training, promotes drug rehabilitation through mind-body regulation and improved physical function.	RCT controlled for confounding factors; for the Chinese male population, the results apply to similar drug rehabilitation groups in exercise rehabilitation intervention studies.

Jin et al. (2025)	Intervention-controlled study	Meth use disorder	Acute aerobic exercise + acute resistance training (single intervention, intensity-matched)	Drug craving intensity, emotional state (anxiety/depression scores), cognitive function (attention/inhibitory control)	Different types of exercise differentially improve craving and emotion-related cognitive factors by regulating neurotransmitter release and activating specific brain regions.	Group-balanced exercise type external variables; generalizable to dependent exercise intervention type selection and mechanism research
